# Could Cytochrome P450 2D6, 3A4 and 3A5 Polymorphisms Explain the Variability in Clinical Response to Clomiphene Citrate of Anovulatory PCOS Women?

**DOI:** 10.3389/fendo.2021.718917

**Published:** 2021-10-08

**Authors:** Camille Robin, Benjamin Hennart, Franck Broly, Philippine Gruchala, Geoffroy Robin, Sophie Catteau-Jonard

**Affiliations:** ^1^ University of Lille, Centre Hospitalier Universitaire (CHU) Lille, Department of Reproductive Medicine, Lille, France; ^2^ University of Lille, CHU Lille, Service de Toxicologie et Génopathies, Pôle de Biochimie et Biologie Moléculaire, Centre de Biologie Pathologie, Lille, France; ^3^ University of Lille, CHU Lille, EA 4308 “Gametogenesis and Gamete Quality”, Lille, France; ^4^ University of Lille, CHU Lille, INSERM U1172, Lille, France

**Keywords:** clomiphene citrate, cytochrome P 450 2D6, anti-Müllerian hormone (AMH), ovulation induction, polycystic ovary syndrome (PCOS)

## Abstract

**Introduction:**

Cytochrome P450 2D6, 3A4 and 3A5 are involved in the metabolism of many drugs. These enzymes have a genetic polymorphism responsible for different metabolic phenotypes. They play a role in the metabolism of clomiphene citrate (CC), which is used to induce ovulation. Response to CC treatment is variable, and no predictive factors have thus far been identified.

**Objective:**

To study a possible link between the cytochrome P450 2D6, 3A4 and 3A5 polymorphisms and clinical response to CC.

**Study Design:**

Seventy-seven women with anovulatory Polycystic Ovarian Syndrome (PCOS) treated with CC were included which determined their cytochrome P450 2D6, 3A4 and 3A5 genotypes and used the results to predict ovarian response to this drug. Predicted responses based on the cytochrome genotypes were compared with the observed clinical responses using the calculation of a weighted Kappa coefficient.

**Main Outcome Measures:**

Number of dominant follicles assessed by ultrasound at the end of the follicular phase and confirmation of ovulation by blood progesterone assay in the luteal phase.

**Results:**

Concordance between the predicted and observed responses for the combination of the three cytochromes was 36.71%, with a negative Kappa coefficient (K = -0.0240), which corresponds to a major disagreement. Similarly, for predictions based on the cytochrome P450 2D6 genotype alone, only 39.24% of predictions were verified (coefficient K = -0.0609).

**Conclusion:**

The genetic polymorphism of cytochromes P450 2D6, 3A4 and 3A5 does not appear to influence clinical response to CC used to induce ovulation in anovulatory PCOS women.

## Introduction

Cytochromes P450 are haemoproteins that play a role in the metabolism of many drugs, mainly in the liver. Fifty-seven genes encoding more than 21,000 P450 cytochromes have been identified (1).

Cytochrome P450 2D6 (CYP2D6) represents only a small percentage — <2% (2) of all cytochromes P450. Nonetheless, its role in drug metabolism is quite important, as it is involved in the metabolism of 20 to 25% of them (3, 4). Its complicated genetic polymorphism (4–9) is responsible for at least four different phenotypes, from ultra-rapid to extensive, intermediate, and poor metabolisers (8, 10).

Various studies have shown that genotyping methods are reliable and can accurately predict different phenotypes and activity levels (scores) of CYP2D6 (4, 11–14). Moreover, its activity depends solely on its genotype and its locus structure, as well as potential drug interactions; it is not influenced by factors such as smoking, caffeine consumption, age, or sex (15, 16). Given this cytochrome’s involvement in the metabolism of so many drugs, it is useful to know patients’ activity scores, to be able to predict the effectiveness of these treatments and adapt their doses. The amounts of the mother-molecule and metabolites appear to depend on the amount and capacity of CYP2D6 to metabolize the specific drug (6). Thus, ultra-rapid CYP2D6 metabolizers (UMs) transform the main active substance too rapidly when the mother-molecule is active, so that therapeutic levels are not reached and drug effectiveness decreases. The reverse occurs for poor metabolizers (PMs): the minimal transformation of mother-molecules into metabolites increases therapeutic effectiveness. In contrast, the effectiveness of prodrugs requiring activation by CYP2D6 is lower in PMs and higher in UMs.

Of the drugs metabolized at least partially by CYP2D6, the metabolism of half is significantly affected by this cytochrome’s genetic polymorphism. These include a large number of antidepressants, antipsychotics, antiarrhythmics and analgesics (4, 6, 7, 17, 18).

At the same time, many publications have studied the association between CYP2D6 and tamoxifen, an anti-oestrogen used in premenopausal breast cancer patients to reduce the risk of recurrence. Most of these studies and recent meta-analyses indicate that the risk of recurrence and disease-free survival rates are lower in patients with a PM-type CYP2D6 phenotype (6, 9, 19–24). Tamoxifen is active through its main metabolite, endoxifen, and the conversion of tamoxifen to endoxifen is performed by CYP2D6. Recommendations for genotyping CYP2D6 before tamoxifen therapy vary by country (25).

The structure of tamoxifen and its metabolism are remarkably similar to those of clomiphene citrate (CC), a treatment belonging to the same pharmacological class (i.e. selective estrogen receptor modulator) and frequently used in the management of anovulatory PCOS (26–28). However, few studies have been published concerning the possibility of predicting response to this treatment according to the patients’ CYP2D6 status.

There is some inter-individual variability in response to CC treatment.Thus, an ovulation is obtained in 73% of cases at doses ranging from 1 to 3 tablets (50 to 150 mg) (29). The predictive factors for resistance to CC are poorly defined, the results of the different studies are contradictory and it is not currently possible to correctly predict the failure of ovulation induction (30–33).

CC is a mixture of two geometric isomers: enclomiphene citrate (E-clomiphene) and zuclomiphene citrate (Z-clomiphene) at a ratio of 62% to 38% (34). E-Clomiphene is mainly metabolised by CYP2D6, and Z-clomiphene by CYP3A4 and CYP3A5 (35–37). In 2012, Mürdter et al. (37) used studies in pooled liver microsomes to describe 9 metabolites of CC. Two of them were very active *in vitro* in antagonism of oestrogen receptors: E-4-hydroxy-N-desethylclomiphene [(E)-4-OH-DE-CLOM] and E-4-hydroxyclomiphene [(E)-4-OH-CLOM], both derived from E-clomiphene (37).

Until now, only four teams have studied the link between the response to CC and the CYP2D6 genotype, and their results have been contradictory (35, 37–39).

The objective of this study was to evaluate the association between the CYP2D6 genotype and clinical response to CC in infertile women with ovulation disorders. The aim of our study is to examine whether the variability of clinical response to this treatment is related to the cytochrome genotype. Secondarily, this study evaluated the association between the combination of genotypes for CYP2D6, CYP3A4 and CYP3A5 and clinical responses to CC.

## Materials And Methods

### Study Design

This retrospective, observational, single-centre study reviewed records of women seen in the endocrine gynaecology department of Lille University Hospital from July 2005 to January 2018 and included 77 women aged 20 to 39 years. All provided written informed consent for the genetic study and data analysis. The ethics committee approved this study (registration number: DGS 2003/0085).

### Population

All women underwent at least one ovulation induction cycle with CC in the endocrine gynaecology department. These women, who wanted to become pregnant, had anovulatory PCOS: they had cycle disorders (oligospaniomenorrhea or amenorrhea) associated with the ultrasound and/or hormonal criteria of the modified Rotterdam consensus definition of PCOS. Other infertility factors (tubal or male) were ruled out. A questionnaire about dietary habits and the use of drugs and herbs was performed.

The hormonal assessment included at least the assay of AMH. Before 2016, serum AMH was measured by manual ELISA using the EIA AMH/MIS kit (A11893 Immunotech, Beckman Coulter, France). The limit of quantification (LoO) of the assay was 2.5 pmol/L. The intra-assay coefficient of variation varies from 3.7%–9.5% for a 151.4- to 5.8-pmol/L concentration range. The inter-assay coefficient of variation varies from 7.8%–21.1% for a 140- to 2.2-pmol/L concentration range. From January 2016, ELISA has been replaced by a fully automated immunoassay on the Access Dxi automatic analyzer (B13127, Beckman Coulter). The LoQ of the assay was 0.57 pmol/L. The within-run and between-run precision varied between 0.9–3.6% and 2.9–10.7%, respectively (40). The dosages of AMH prior to January 2016 were recalculated to compare them to with those performed in 2018 and after. The conversion formule used was « AMH (dxi) = 0,77 x AMH (ELISA) + 0,12 », in pmol/L, as previously shown (41). Pelvic ultrasound was performed vaginally with a 5-9 Mhz probe and a Voluson E8 Expert (General Electric Systems, Velizy, France).

### Study Protocol

For the first induction cycle of ovulation, women received 50 mg/day of CC from the second to the sixth day of the menstrual cycle (spontaneous or triggered by dydrogesterone for women with amenorrhoea). A pelvic ultrasound was performed between the tenth and twelfth day of the cycle to measure the diameter of the dominant follicle(s). If there was no follicle larger than 10 mm in diameter, a new ultrasound was performed 7 days later. The progesterone level was measured 8 to 10 days after the expected date of ovulation. Women with a progesterone level greater than 5 ng/ml in the luteal phase with growth of one or two follicles larger than 10 mm on ultrasound at the end of the follicular phase were considered to respond to 1 tablet of CC and remained at this dose for subsequent cycles in the absence of pregnancy, with the same monitoring. If progesteronaemia was lower than 5 ng/ml, the CC dosage was increased to 100 mg/day in the following cycle, and up to 150 mg/day if there was still no response. All women underwent one to six cycles of ovulation induction with CC. If more than two dominant follicles grew, the response was considered too strong, protected sexual relations were recommended, and the women received 25 mg/day during the next cycle.

Four groups of observed clinical response were described:

-increased, after response by more than two dominant follicles at 50 mg/day,
-normal, after response by one or two dominant follicles at 50 or 100 mg/day,
decreased, after response by one or two dominant follicles at 150 mg/day,
-null/nearly-zero when there remained no response at 150 mg/day.

A sample of two EDTA tubes of 5 ml of peripheral blood was taken from all patients during one of their visits for consultation in the department, for genetic analysis.

### CYP2D6, CYP3A4 and CYP3A5 Genotyping

The DNA extraction was performed on a CheMagic Star (Hamilton Robotics) integrated system according to the supplier’s instructions. The CYP2D6 gene was sequenced by the BigDye Terminator Kit sequencing kit (Applied Biosystems Ltd); electrophoresis and analysis of the reaction products were performed on an Applied Biosystems 3130 XL 48 automated capillary sequencer. Sequences were analyzed with SeqScape Software v2.5.6. The reference cDNA sequence used was NM_000106.5, with the A of translation initiation ATG numbered as 1. The search for structural rearrangements of the CYP2D6 gene locus, with deletion type and amplification, was carried out with the TaqMan kit, Copy Number Assay ID Hs00010001_cn marketed by Applied Biosystems (Applied Biosystems, Foster City, CA, USA), and a high-speed controller (TaqMan 7900, Applied Biosystems), according to the suppliers’ instructions.

To genotype CYP3A4 and CYP3A5, mutations (NM_017460.5: c522-191C> T (rs35599367) for CYP3A4 and NM_000777.4: c219-237A> G (rs776746) for CYP3A5) were sought with a method based on real-time PCR, high-throughput PLC (TaqMan 7900, Applied Biosystems) and the use of a TaqMan SNP Genotyping Assay Kit (Assay ID: C_59013445_10, Applied Biosystems for CYP3A4 and Assay ID: C_26201809_30, Applied Biosystems for CYP3A5). We arbitrarily considered that an allele of the gene not carrying the mutation was functional (CYP3A4 * 1, CYP3A5 * 1).

### Prediction of Responses According to CYP2D6, CYP3A4 and CYP3A5 Genotypes

For the metabolic pathway mediated by CYP2D6, enabling the synthesis of the most active metabolites (37): The women were classified into four groups: PMs (poor metabolisers), UMs (ultra-rapid metabolisers), IMs (intermediate metabolisers), and EMs (extensive metabolisers), in accordance with the references previously cited (8, 10).

For the analysis of the concordance of predicted responses according to the CYP2D6 genotype and the actual response, PMs were considered to have an expected null or near zero response, IMs a weak response, EMs a normal response, and UMs a strong response.

For the metabolic pathway mediated by CYP3A4 and CYP3A5 for the synthesis of Z-desethylclomiphene:

Four activity groups were created:

-Limited when the genotype results in CYP3A4 and CYP3A5 deficiency,
-Intermediate1 when the genotype results in CYP3A5 deficiency with normal CYP3A4 activity,
-Intermediate 2 when the genotype results in CYP3A4 deficiency with normal CYP3A5 activity,
-Extensive when the genotype results in functional CYP3A4 and CYP3A5.

Combining the predictions of activity of the two metabolic pathways allowed us to predict the overall response to CC, as summarized in **Table 1**. For example, in the case of mutations in CYP3A4 and CYP3A5, more mother molecules (CC) go to the CYP2D6 pathway, so the overall activity is increased.

**Table 1:** Prediction of response to clomiphene citrate according to CYP 2D6, CYP 3A4 and CYP 3A5 phenotypes.

Phenotype 2D6	UM	EM	IM	PM
Phenotype 3A4 + 3A5			
Extensive : functional + functional	Normal or Increased	Low	Low	Nearly zero
Intermediate1 Functional+ Deficit	Increased	Normal	Possibly decreased	Low
Intermediate2 Deficit + Functional	Increased	Normal	Possibly decreased	Low
Limited: Deficit + Deficit	Increased	Increased	Normal	Low

UM, Ultra-fast Metabolizer; EM, Extensive Metabolizer; IM, Intermediate Metabolizer; PM, Poor Metabolizer.

### Statistical Analysis

Patients’ characteristics according to their observed clinical response and CYP2D6 genotype were compared by the Kruskal-Wallis test, with the Statistical Package for the Social Sciences software (SPSS, Chicago II USA, version 22.0).

The calculation of a weighted Kappa coefficient made it possible to evaluate the correlation between the observed clinical responses and the predicted responses according to either the CYP2D6 genotype only or the combination of the CYP2D6, CYP3A4 and CYP3A5 genotypes.

The qualitative indicators are described by frequency and percentage.

A Kappa coefficient greater than 0.8 corresponds to strong agreement between the two indicators, a coefficient between 0.8 and 0.4 to moderate agreement, a coefficient below 0.4 to weak agreement, and a negative Kappa coefficient to no agreement (beyond that due to chance).

These coefficients were calculated by the Biostatistics Methodology Unit of Lille University Hospital, with SAS software (SAS Institute version 9.4). In a second step, a *post hoc* ANOVA Bonferroni correction was performed with SPSS software to compare the AMH values of the different clinical response groups.

## Results

**Figure 1:**
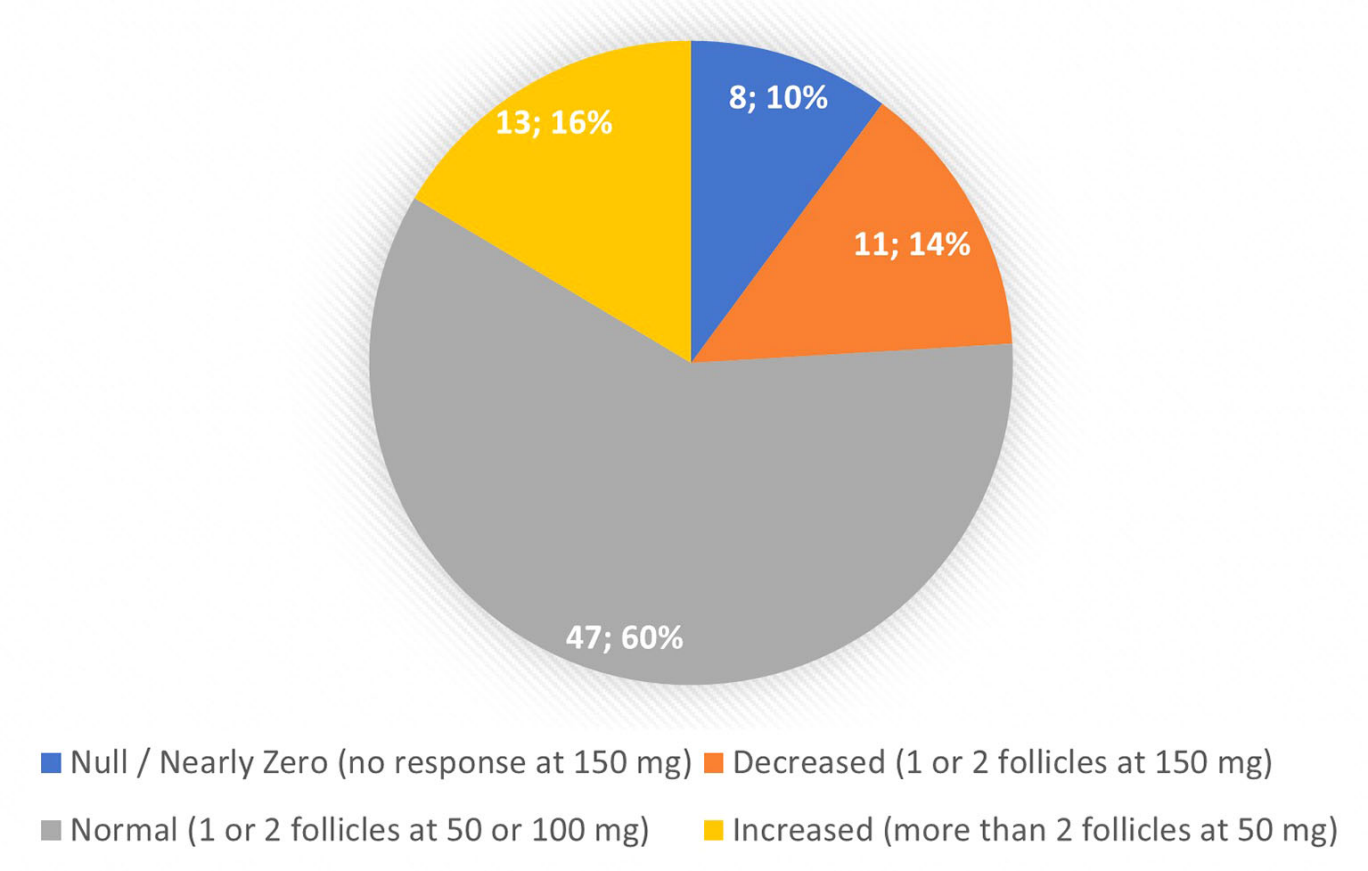
Clinical Observed Responses.

The study included 77 women, two managed in our department for two different pregnancies. Accordingly, 79 clinical response profiles were observed. **Table 2** presents the women’s general characteristics. Four women had PM phenotypes predicted according to their genotype, including one patient with a homozygous deletion of the CYP2D6 gene. In contrast, 5 women had predicted UM phenotypes, 41 normal (EM) and 27 IM. Clinical responses, on the other hand, showed 8 null or near-zero responses, 11 low, 47 normal, and 13 high (**Figure 1**).

**Table 2:** Demographic characteristics.

Characteristics	Median [5^ème^-95^ème^percentiles]
**Age (years)**	28 [21-36]
**BMI (kg/m^2^)**	25 [18.5-35.4]
**AMH (pmol/L)**	72.8 [26-190]
**AFC**	55.5 [25-135]

**Tables 3** and **4** present patient characteristics according to clinical response group and to CYP2D6 genotype, respectively. The Kruskal-Wallis test found no significant differences in age (*P* = 0.900), BMI (*P* = 0.715) or antral follicle count (AFC) (*P* = 0.051), while responses were better with lower AMH concentrations (*P* = 0.020). At the same time, the CYP2D6 phenotypes did not differ significantly for the four variables studied.

**Table 3:** Characteristics of the patients according to clinical observed response group.

		Clinical Observed Response				
		Null/Nearly-zero ^1^(n=8)	Decreased ^2^(n=11)	Normal ^3^(n=47)	Increased ^4^(n=13)	*p* value
**Characteristics [median** **(5^ème^-95^ème^ percentiles)]**	**Age (years)**	27.5 [21-32]	28 [22-34]	28 [20.4-36]	29 [23-35.6]	**0.900**
	**BMI (kg/m^2^)**	27,8 [18.4-35.7]	27 [19-33.4]	25,2 [19-35.85]	24 [18.5-32.1]	**0.715**
	**AMH** **(pmol/L)**	130.15 [63-115.2]	84.8 [35-111.8]	72.15 [22.4-185]	51,25 [35.8-118.5]	**0.020**
	**AFC**	81.5 [33-115.2]	75 [29-114.1]	54 [17.8-93]	47 [30-136.2]	**0.051**

^1^: no response at 150 mg; ^2^: 1 or 2 follicles at 150 mg; ^3^: 1 or 2 follicles at 50 or 100 mg; ^4^: > 2 follicles at 50 mg.

Data in bold are p-values and are non-significant (NS) if greater than or equal to 0.05.

**Table 4:** Characteristics of the patients according to CYP2D6 status.

		CYP2D6 Status				
		PM (n=4)	IM (n=27)	EM (n=41)	UM (n=5)	*p* value
**Characteristics** **[median** **(5^ème^-95^èee^ percentiles)]**	**Age (years)**	25 [20-32]	29 [22.45-35.55]	28 [21-37.7]	26 [24-27]	**0.083**
	**BMI (kg/m^2^)**	23.25 [19-30]	25.4 [18.5-35.7]	24.6 [18.49-36.36]	30.8 [22.8-34]	**0.521**
	**AMH** **(pmol/L)**	59.75 [40.6-170]	84.45 [19.33-190]	67.5 [29.18-198.5]	84.8 [72.8-126]	**0.743**
	**AFC**	65.5 [47-82]	59.5 [13.9-135]	54 [26-167]	57.5 [38-66]	**0.872**

PM, Poor Metabolizer; UM, Ultra-fast Metabolizer; IM, Intermediate Metabolizer; EM, Extensive Metabolizer.

Data in bold are p-values and are non-significant (NS) if greater than or equal to 0.05.

Analysis of the expected responses according to CYP2D6 genotype and observed response showed a negative Kappa coefficient (K = -0.0609), which corresponds to a major discrepancy between the observed clinical responses and the responses expected according to CYP2D6 genotype (**Table 5**). Only 39.24% of the predicted responses were clinically verified.

**Table 5:** Clinical responses observed according to CYP2D6 status.

		CYP2D6 Phenotype (Response Prediction)				Total
		PM (Null/Nearly-zero)	IM (Decreased)	EM (Normal)	UM (Increased)	
**Clinical Responses Observed**	**Null/Nearly-zero ^1^**	0 0.00%	1 1.27%	7 8.86%	0 0.00%	**8** **10.13%**
	**Decreased ^2^**	0 0.00%	6 7.59%	4 5.06%	1 1.27%	**11** **13.92%**
	**Normal ^3^**	2 2.53%	16 20.25%	25 31.65%	4 5.06%	**47** **59.49%**
	**Increased ^4^**	2 2.53%	5 6.33%	6 7.59%	0 0.00%	**13** **16.46%**
**Total**		4 5.06%	28 35.44%	42 53.16%	5 6.33%	**79** **100.00%**
**Coefficient of concordance Kappa: K= -0.0609**						

PM, Poor Metabolizer; UM, Ultra-fast Metabolizer; IM, Intermediate Metabolizer; EM, Extensive Metabolizer.

^1^: no response at 150 mg; ^2^: 1 or 2 follicles at 150 mg; ^3^: 1 or 2 follicles at 50 or 100 mg; ^4^: > 2 follicles at 50 mg.

Data in bold are the frequencies and percentages of the population.

Similarly, the Kappa coefficient for agreement between the expected responses according to the combination of the genotypes of the 3 cytochromes and the observed responses was negative (K = -0.0240) (**Table 6**), with 36.71% concordance between predictions and actual responses.

**Table 6:** Clinical observed responses according to response predictions with the combination of CYP2D6, CYP3A4 and CYP3A5 status.

		Expected Responses				Total
		Null/Nearly-zero^1^	Decreased ^2^	Normal ^3^	Increased ^4^	
**Clinical responses observed**	**Null/Nearly-zero ^1^**	0 0.00%	2 2.53%	6 7.59%	0 0.00%	**8** **10.13%**
	**Decreased ^2^**	0 0.00%	8 10.13%	2 2.53%	1 1.27%	**11** **13.92%**
	**Normal ^3^**	0 0.00%	21 26.58%	22 27.85%	4 5.06%	**47** **59.49%**
	**Increased ^4^**	1 1.27%	8 10.13%	4 5.06%	0 0.00%	**13** **16.46%**
**Total**		**1** **1.27%**	**40** **50.63%**	**33** **41.77%**	**5** **6.33%**	**79** **100.00%**
**Coefficient of concordance Kappa : K= -0.0240**						

^1^: no response at 150 mg; ^2^: 1 or 2 follicles at 150 mg; ^3^: 1 or 2 follicles at 50 or 100 mg; ^4^: > 2 follicles at 50 mg.

Data in bold are the frequencies and percentages of the population.

Finally, the *post hoc* ANOVA Bonferroni correction was performed to determine if AMH was predictive of the clinical response to CC. Lack of response was significantly associated with higher AMH levels than for women who responded weakly, normally, or excessively (*P* = 0.040, *P* = 0.06, and *P* = 0.02 respectively). Results for the other categories of clinical response were not significant (data not shown).

## Discussion

This study shows that the CYP2D6 genotype does not correlate with clinical response to CC in anovulatory PCOS women. According to our extensive review of the available literature, this is only the second study to compare clinical response to CC by cytochrome P450 2D6 genotype.

Our results are in agreement with those of the team of Ji et al. (38); they observed no correlation between the CYP2D6 genotype and either the clinical response or active metabolite concentrations. Their study also showed a correlation between the clinical response to CC and plasma concentrations of the parent and active metabolites: the plasma concentrations of E-clomiphene were significantly higher in the responder group. There was a trend towards an increase in the active (E)-4-OH-Clom and (E)-4-OH-DE-Clom (NS) metabolites, compared to the non-responder patients, who had significantly higher (Z)-clomiphene-NO concentrations.

Similarly, in a recent study, Kim et al. (39) found higher concentrations of active metabolites in women with CYP2D6 mutations causing decreased CYP2D6 activity. Their results disagreed with those of Mürdter et al. (37), who reported a correlation between genotype and plasma concentrations. Plasma levels of the (E)-4-OH-Clom and (E)-4-OH-DE-Clom active metabolites were 8 to 50 times higher in their four extensive metabolizer (EM) patients than in the two (PM) women, who had parent drug concentrations (E-clomiphene) 6 times higher. These results are consistent with those of Ghobadi et al. (35) in 2008, who claimed that the extent of E-clomiphene metabolism was correlated with the amount of CYP2D6 present.

Nevertheless, neither of these studies evaluated the clinical response in ovulation induction. Furthermore, in 2009, Ghobadi et al. (34) reported that E-clomiphene and Z-clomiphene concentrations did not predict clinical response to CC in inducing ovulation, a finding inconsistent with those of the two previous studies and of those by Ji et al. (38).

One of the strengths of our population is the presence of “extreme” phenotypes, with 4 women PM for CYP2D6, 5 UM, and 4 with high levels of CYP3A4 and CYP3A5. This was not the case in the Korean population studied by Ji’s team (38), which failed to recruit any PM or UM phenotypes. Nevertheless, these numbers remain low and result in a lack of power for our study. It is therefore an initial finding, the results of which must be confirmed by other studies with higher numbers.

One hypothesis that might explain our results is that the women were taking other drugs metabolized by CYP2D6, which could provoke competitive CYP2D6 activity and modify metabolism and thus clinical responses to therapeutics. Women were asked about other drugs, however, and reported taking only folic acid and CC.

The clinical response to CC does not appear to be directly correlated with the combination of women’s CYP2D6, CYP3A4 and CYP3A5 genotypes. The observation of two totally different clinical responses for the same patient reinforces this statement. Of the two women who underwent stimulation at two different periods for a second pregnancy, one responded normally for the first stimulation, and not at all the second time. The only factors that were changed between her two stimulations were age (+4 years) and 10 kg (BMI increased from 28 to 31.4), but age and BMI were not significantly associated with a poorer response to CC in our study (*P* = 0.900 and *P* = 0.715, respectively). Other interacting factors probably explain the differences in clinical response to this treatment. As previously reported (42), most of the therapeutic effects of drugs are related to complex interactions between genetic, clinical, biological and environmental factors.

So far, although numerous studies have been looking for factors predicting response to CC (30–33), no factors have been found to accurately predict women’s capacity to respond to it. Our results are consistent with these studies with respect to age, BMI and AFC. However, the clinical response to CC in our cohort appeared to be better when the AMH concentration was not too high (*P* = 0.020).

*Post hoc* ANOVA analysis with the Bonferroni correction found that AMH levels were significantly higher in the group of women who did not respond clinically to CC, but results for the other categories of clinical response were not significant. In addition, the woman who was stimulated twice, four years apart, and did not respond to the second stimulation had no significant change in her AMH level (63 pmol/l *vs* 68 pmol/l at the first stimulation). These observations led us look for a possible difference in AMH levels according to CYP2D6 phenotypes, but we found no difference (*P* = 0.743). The CYP2D6 genotype does not seem to determine the AMH level.

Other mechanisms may also explain our results. The activity of CC may be mediated not only by the metabolites, but also by the parent molecule. There are probably other mutations of CYP2D6, CYP3A4 and CYP3A5, not known at this time, that modify the predicted phenotypes, as others have pointed out (24). For example, we looked for only one mutation for each of CYP3A4 and CYP3A5, using the TaqMan technique. On the other hand, for CYP2D6, the Sanger sequencing technique used in our study can detect a large number of mutations, and it seems unlikely that known mutations were not found. The metabolism of CC may be different from that assumed by Mürdter (37): Mazzarino and colleagues (36) described a CC metabolism different from that defined by Mürdter with *in vitro* analyses the previous year (37). Mazzarino’s team (36) described 22 metabolites *in vivo* instead of 9 and found only 4 of the 9 metabolites previously mentioned. Moreover, they found a different level of CYP2D6 activity for this metabolism, which might modify the predicted responses. Accordingly, a better understanding of the metabolism of CC and the activity of different cytochromes involved in it appears necessary before any more powerful study of clinical response can be conducted. It would be useful for these future studies to include measurements of the plasma concentrations of CC metabolites during the stimulation cycle.

In conclusion, the genetic polymorphism of cytochromes P450 2D6, 3A4 and 3A5 does not seem to predict the clinical response to CC in our population of 77 anovulatory PCOS women. However, our study’s low power requires confirmation of the results by larger studies to define the predictors of response to this ovulation inducer. A more precise knowledge of the metabolism of this drug and its relation to cytochrome P450 is necessary.

## Data Availability Statement

The raw data supporting the conclusions of this article will be made available by the authors, without undue reservation.

## Ethics Statement

This retrospective, observational, single-centre study reviewed records of women seen in the endocrine gynaecology department of Lille University Hospital from July 2005 to January 2018 and included 77 women aged 20 to 39 years. All provided written informed consent for the genetic study and data analysis. The ethics committee approved this study (registration number: DGS 2003/0085).

## Author Contributions

Each author acknowledges that he or she participated sufficiently in the work to take public responsibility for its content. All authors contributed to the article and approved the submitted version.

## Conflict of Interest

The authors declare that the research was conducted in the absence of any commercial or financial relationships that could be construed as a potential conflict of interest.

## Publisher’s Note

All claims expressed in this article are solely those of the authors and do not necessarily represent those of their affiliated organizations, or those of the publisher, the editors and the reviewers. Any product that may be evaluated in this article, or claim that may be made by its manufacturer, is not guaranteed or endorsed by the publisher.
